# The Wilcox Bill

**Published:** 1892-07

**Authors:** 


### THE WILCOX BILL.

Especial attention is called to the statement issued by the Maryland State Dental Society upon this bill, which has recently passed the House of Representatives.

The interest taken in this matter by the dentists of Maryland is not only worthy of unstinted praise, but they should receive the cordial and immediate support of the profession throughout the country. This must be given at once if anything is to be accomplished. Let each one make it his special business to write to the senators of his State. Flood them with letters and petitions, and in this way create a sentiment of opposition.

While statistics of mechanical work connected with dentistry would unquestionably be of great value, provided they were correct, the means adopted by the Census Bureau have been of such a character that self-respecting dentists have, in many instances, refused to answer. This bill makes this imperative, and virtually places dentists outside the pale of the professions. Are the dentists of the country willing to permit this bill to become a law, without an effort to defeat it? The answer to this must be in immediate work.

